# Enhanced Bacterial α(2,6)-Sialyltransferase Reaction through an Inhibition of Its Inherent Sialidase Activity by Dephosphorylation of Cytidine-5'-Monophosphate

**DOI:** 10.1371/journal.pone.0133739

**Published:** 2015-07-31

**Authors:** Ji-Yeon Kang, Se-Jong Lim, Ohsuk Kwon, Seung-Goo Lee, Ha Hyung Kim, Doo-Byoung Oh

**Affiliations:** 1 Synthetic Biology and Bioengineering Research Center, Korea Research Institute of Bioscience & Biotechnology (KRIBB), Daejeon, Korea; 2 Biosystems and Bioengineering Program, University of Science and Technology (UST), Daejeon, Korea; 3 College of Pharmacy, Chung-Ang University, Seoul, Korea; Universidade de São Paulo, BRAZIL

## Abstract

Bacterial α(2,6)-sialyltransferases (STs) from *Photobacterium damsela*, *Photobacterium* sp. JT-ISH-224, and *P*. *leiognathi* JT-SHIZ-145 were recombinantly expressed in *Escherichia coli* and their ST activities were compared directly using a galactosylated bi-antennary *N*-glycan as an acceptor substrate. In all ST reactions, there was an increase of sialylated glycans at shorter reaction times and later a decrease in prolonged reactions, which is related with the inherent sialidase activities of bacterial STs. These sialidase activities are greatly increased by free cytidine monophosphate (CMP) generated from a donor substrate CMP-*N*-acetylneuraminic acid (CMP-Neu5Ac) during the ST reactions. The decrease of sialylated glycans in prolonged ST reaction was prevented through an inhibition of sialidase activity by simple treatment of alkaline phosphatase (AP), which dephosphorylates CMP to cytidine. Through supplemental additions of AP and CMP-Neu5Ac to the reaction using the recombinant α(2,6)-ST from *P*. *leiognathi* JT-SHIZ-145 (P145-ST), the content of bi-sialylated *N*-glycan increased up to ~98% without any decrease in prolonged reactions. This optimized P145-ST reaction was applied successfully for α(2,6)-sialylation of asialofetuin, and this resulted in a large increase in the populations of multi-sialylated *N*-glycans compared with the reaction without addition of AP and CMP-Neu5Ac. These results suggest that the optimized reaction using the recombinant P145-ST readily expressed from *E*. *coli* has a promise for economic glycan synthesis and glyco-conjugate remodeling.

## Introduction

Sialic acid (SA) represents a family of negatively charged mononucleotides with nine-carbon backbones that include the most common *N*-acetylneuraminic acid (Neu5Ac) and its derivatives. It usually locates at the non-reducing terminal of glycans with four linkage types to penultimate residues [Neu5Ac-α(2,3)-Gal, Neu5Ac-α(2,6)-Gal, Neu5Ac-α(2,6)-GalNAc, and Neu5Ac-α(2,8)-Neu5Ac]. In *N*-glycans of glycoproteins, Neu5Ac-α(2,3)-Gal [α(2,3)-SA] and Neu5Ac-α(2,6)-Gal [α(2,6)-SA] are most commonly found. This SA capping of galactose residues is important for prolonged *in vivo* half-life of glycoproteins; exposed galactose is recognized by the Ashwell receptor in the liver, which removes the asialoglycoproteins from the circulation [1]. Many efforts have been made to ensure homogeneous SA capping in the manufacturing of therapeutic glycoproteins because non-sialylated glycoproteins do not show effective *in vivo* efficacies owing to a short half-life. While both α(2,3)- and α(2,6)-SA are important for *in vivo* half-life, it has been elucidated that there are several different biological and pathophysiological roles which are determined by their specific linkages. Influenza virus to infect humans binds preferentially to an α(2,6)-SA ligand on the surface of host cells whereas avian influenza viruses prefer the α(2,3)-SA linkage [2]. *N*-glycan attached to Asn297 of immunoglobulin G (IgG) heavy chain is known to be a signal for pro- or anti-inflammatory response. Especially, the addition of α(2,6)-SA to the glycan of the IgG Fc fragment using rat α(2,6)-sialyltransferase (ST) mediated anti-inflammatory response in a mouse rheumatoid arthritis model whereas α(2,3)-sialylation showed no effect [3]. Also, it has been reported that a large amount of recombinant human α(2,6)-sialyltransferase (hST6Gal1) produced from HEK293F cells is required for the *in vitro* reaction to generate bi-α(2,6)-sialylated glycan attached to Fc [4].

For the generation of homogeneously α(2,6)-sialylated glycoproteins, *in vitro* enzymatic reactions have been generally used because Chinese hamster ovary (CHO) cells, the most widely used mammalian cells, express only α(2,3)-ST while human cells express both α(2,3)- and α(2,6)-STs [5]. The α(2,6)-sialylations were carried out using eukaryotic α(2,6)-STs, either purified from mammalian tissues or recombinantly produced from insect or mammalian cell culture; it has been known that eukaryotic α(2,6)-STs are not well expressed in bacterial systems as soluble and functionally active forms [4,6]. It would be more convenient and economical to use a bacterial ST recombinantly produced from *Escherichia coli* because bacterial enzymes are more soluble and functionally active in *E*. *coli* expression. Although many bacterial STs have been found and characterized for α(2,3)-sialylation, only four α(2,6)-STs have been reported, from *Photobacterium damsela*, *P*. *leiognathi* JT-SHIZ-145 and JT-SHIZ-119, and *Photobacterium* sp. strain JT-ISH-224 [7–10]. All of them belong to one subgroup of glycosyltransferase family 80 in the CAZy (carbohydrate-active enzymes) database.

In this study, we recombinantly expressed three bacterial α(2,6)-STs in *E*. *coli* and compared their ST activities using a galactosylated bi-antennary *N*-glycan (G2; Gal_2_Man_3_GlcNAc_4_) and cytidine-5`-monophosphate (CMP)-Neu5Ac as acceptor and donor substrates respectively. During biochemical analysis, all of the tested STs were found to display sialidase activity, which was induced by CMP released from CMP-Neu5Ac during the ST reaction. We showed that the removal of free CMP by simple treatment of alkaline phosphatase (AP) and supplemental addition of CMP-Neu5Ac greatly improve the α(2,6)-ST reaction.

## Materials and Methods

### Materials

Cytidine 5′-diphosphate (CDP), cytidine, adenosine 5′-diphosphate (ADP), and 6`-sialyllactose were purchased from GeneChem (Daejeon, Korea). Peptide-N-glycosidase F (PNGase F) and graphitized carbon column were obtained from New England Biolabs (Ipswich, MA, USA) and Alltech (Lexington, MA, USA) respectively. Asialo, galactosylated, biantennary oligosaccharide (G2 glycan), calf intestinal alkaline phosphatase, and biotinylated *Sambucus nigra* (SNA) lectin were purchased from Prozyme (Hayward, CA), Takara (Tokyo, Japan), and Ey Laboratories (San Mateo, CA, USA) respectively. Solvents for high-performance liquid chromatography (HPLC) including acetonitrile and water were purchased from Burdick and Jackson (Muskegon, MI, USA). 2-Aminobenzoic acid (AA), sodium cyanoborohydride, acetic acid, tetrahydrofuran, triethylamine, trifluoroacetic acid (TFA), CMP-NeuAc, adenosine 5′-triphosphate (ATP), cytidine 5′-triphosphate (CTP), cytidine 5′-monophosphate (CMP), GDP-galactose, bovine β(1,4)-galactosyltransferase, asialofetuin and other reagents (unless stated otherwise) were purchased from Sigma-Aldrich (St. Louis, MO, USA).

### Cloning, expression, and purification of recombinant α(2,6)-STs

Bacterial α(2,6)-ST genes (Pd-, P224-, and P145-ST) were synthesized with codon optimizations for recombinant expressions in *E*. *coli* by Bioneer gene synthesis service (Daejeon, Korea). From the synthesized genes, the corresponding DNA fragments were amplified by polymerase chain reaction (PCR) in a 50 μl reaction mixture containing 20 pmol of each primer, the template plasmid DNA (10 ng), 1 μl of dNTP mix (10 mM), 0.5 μl of Pfu-X polymerase (2.5 units), and Pfu-X buffer. The used PCR primers are summarized in S1 Table. The Pd-ST PCR products were digested with *BamHI* and *EcoRI*, while the P224- and P145-ST products were digested with *BamHI* and *HindIII*. The purified DNA fragments were cloned into the *BamHI*/*EcoRI* or *BamHI*/*HindIII* restriction site of pColdII vector (Takara). The correct cloning was confirmed by sequencing. The DNA and amino acid sequences of the recombinant proteins expressed by pColdII-PdST,-P224ST, and–P145ST are represented in S1–S3 Figs. These plasmid were transformed into *E*. *coli* BL21 strain for protein expression. A single transformant grown in Luria-Bertani (LB) broth agar plate containing ampicillin (100 μg/ml) was inoculated into Terrific broth (TB) containing 100 μg/ml ampicillin. After growth at 37°C until optical density at 600 nm (OD_600_) reached 0.6–0.8, protein expressions were induced by adding 1.0 mM isopropyl-β-D-thiogalactopyranoside (IPTG) at 15°C for 24 h. The harvested cell pellets were suspended in 50 mM sodium phosphate buffer (pH 8.0) containing 300 mM NaCl, 1 mM PMSF, and 0.1 mg/ml lysozyme, and then lysed by sonication on ice. After the lysate was centrifuged at 7,000 g for 20 min, the supernatant was collected and the recombinant α(2,6)-STs were purified by Ni-NTA affinity chromatography as described previously [11]. The fractions collected during the purification process were analyzed by sodium dodecyl sulphate polyacrylamide gel electrophoresis (SDS-PAGE).

### Sialyltransferase activity assay

G2 glycan was labeled with AA for detection in HPLC as previously described [12], and then used as a substrate to measure the sialyltransferase activity. The reaction mixture consisted of 100 mM Tris-HCl (pH 8.0), 0.5 mM CMP-Neu5Ac, 1 μM AA-labeled G2 glycan, and 1 μg of the purified α(2,6)-ST in 20 μl total volume, and this was incubated for 30 min at 30°C unless stated otherwise: for the time-course of enzyme reactions, incubation times were 10, 30, 60, 120, 240, and 1,440 min. After the termination of reactions by boiling for 5 min, the formations of sialylated glycans were analyzed by HPLC as previously described [12]. Briefly, aliquots of the terminated reaction solution were loaded onto a Shodex Asaipak NH2P-50 (5 μm, 4.6 mm x 250 mm) amine column (Showa Denko, Tokyo, Japan) on a Waters Alliance system equipped with a Waters 2475 fluorescence detector (Milford, MA). The solvent system consisted of two eluents, A (acetonitrile containing 2% acetic acid and 1% tetrahydrofuran) and B (5% acetic acid, 1% tetrahydrofuran, and 3% triethylamine in water). The column was eluted at a flow rate of 1 ml/min with a linear gradient (30–100% solvent B) for 30 min. The fluorescence of AA-labeled glycans was monitored with excitation at 360 nm and emission at 425 nm. Steady-state kinetic parameters were obtained from the sialyltransferase reactions containing 0.1 μg of the purified α(2,6)-STs with 0.5 mM CMP-Neu5Ac and various concentrations of AA-labeled G2 glycan in 20 μl of 100 mM Tris-HCl (pH 8.0). The reaction mixtures were incubated for 5 min at 30°C. The kinetic parameters (*K*_m_ and *V*_max_) were obtained by fitting the data to the Michaelis-Menten equation using nonlinear regression analysis with GraphPad Prism software (GraphPad Software, Inc. San Diego, CA).

### Sialidase activity assay

Sialidase activities of α(2,6)-STs were measured by analyzing the conversions of AA-labeled 6’-sialyllactose to lactose. Reactions were performed in a total volume of 20 μl containing 100 mM sodium phosphate buffer (pH 6.0), 50 μM AA-labeled 6’-sialyllactose, and 2 μg of the purified α(2,6)-ST. They were incubated for 2 h at 37°C and terminated by boiling for 5 min. The formations of AA-labeled lactose were analyzed by HPLC using the same conditions described in the sialyltransferase activity assay. To determine the effects of nucleotides on the sialidase activity, 1.0 mM of CMP, CDP, CTP, cytidine, ADP, or ATP was added to the reaction mixtures unless stated otherwise: for the concentration dependent analysis, 0.01, 0.05, 0.1, 0.5, 1 and 10 mMs of either CMP or cytidine were used. Inhibition of α(2,6)-sialidase activity by the addition of alkaline phosphatase (AP) was also analyzed. The reaction conditions were the same as described above except for the addition of 1.0 mM CMP and 0.1–5 U/μl AP.

### Optimization of sialyltransferase reaction condition

To find the optimized P145-ST reaction condition, several conditions were compared. Basic reaction mixture comprised 100 mM Tris-HCl (pH 8.0), 0.5 mM CMP-Neu5Ac, 1 μM G2 glycan, and 1 μg of P145-ST in a total 20 μl volume, which was incubated for 4 h at 30°C. To enhance sialylation, 1 μl of 10 mM CMP-Neu5Ac and/or 2 μl of 4 U/μl AP were added to the basic reaction solution 30 min after the beginning of the reaction. For further enhancement, 1 μl of 10 mM CMP-Neu5Ac and 2 μl of 4 U/μl AP were added to basic reaction solution two times at 30 and 120 min.

For *in vitro* sialylation of asialofetuin, 100 μg of asialofetuin was used instead of 1 μM G2 glycan in 20 μl of the basic reaction mixture. This was incubated for 4 h at 30°C with and without the two sequential additions of CMP-Neu5Ac (1 μl, 10 mM) and AP (2 μl of 4 U/μl) at 30 and 120 min of the reaction.

### Lectin blot analysis

Additions of α(2,6)-SA to asialofetuin by the P145-ST reaction were analyzed by lectin blot using SNA lectin as previously described [13]. Briefly, two μg of protein were subjected to 10% SDS-PAGE and then transferred to a PVDF membrane. This membrane was treated with biotinylated-SNA lectin (0.8 μg/ml), followed by incubation with a horseradish peroxidase-conjugated streptavidin as a secondary binding reagent.

### *N*-glycan analysis

*N*-glycans of asialofetuin and sialylated fetuins were analyzed using HPLC and matrix-assisted laser-desorption/ionization time-of-flight (MALDI-TOF) mass spectrometry as described previously [12]. Briefly, *N*-glycans were released by PNGase F treatment and purified by solid-phase extraction using graphitized carbon. After labelling with AA, they were analyzed using a Shodex Asaipak NH2P-50 column as described for the sialyltransferase activity assay with a slight modification in elution condition. Elution was carried out at a flow rate of 1 ml/min with a linear gradient (20–70% solvent B) for 70 min. AA-labelled glycans were analyzed in linear negative ion mode using a Microflex (Bruker Daltonik, GmbH, Bremen, Germany) mass spectrometer.

## Results

### Recombinant expression of α(2,6)-STs in E. coli

**Fig 1:**
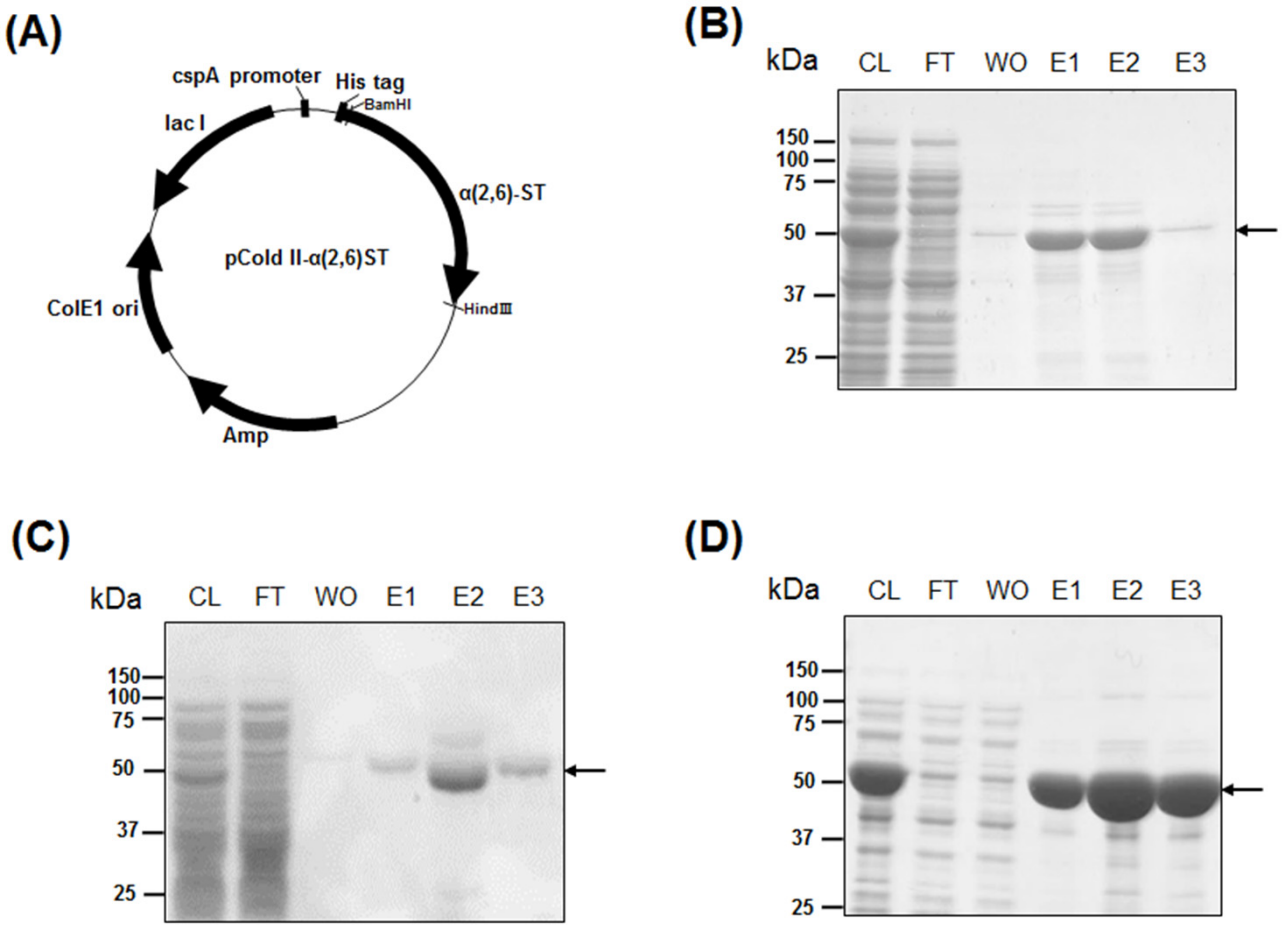
Expression and purification of recombinant α(2,6)-STs in *E*. *coli*. Bacterial α(2,6)-ST genes (Pd-ST, P224-ST, and P145-ST) were cloned into a pCold II vector for recombinant expression in *E*. *coli* (A). Recombinant Pd-ST (B), P224-ST (C), and P145-ST (D) were purified using His-tag affinity chromatography and analyzed using SDS-PAGE. CL: soluble fraction of cell lysate, FT: flow-through fraction, WO: wash-out fraction, E1-3: eluted fractions. Purified α(2,6)-ST protein bands are indicated by the arrows.

The sequences of gene encoding α(2,6)-STs of *P*. *damsela* (accession number BAA25316), *Photobacterium* sp. strain JT-ISH-224 (accession number BAF92026), and *P*. *leiognathi* JT-SHIZ-145 (accession number AB306315) were obtained from the National Center for Biotechnology Information (NCBI). The coding regions including catalytic domains were synthesized with codon optimization for *E*. *coli* expression; 16–497 amino acids of *P*. *damsela* α(2,6)-STs [7], 18–514 amino acids of *Photobacterium* sp. strain JT-ISH-224 [10], and 16–497 amino acids of *P*. *leiognathi* JT-SHIZ-145 [8] were designated as Pd-, P224-, and P145-ST respectively. The synthesized DNA fragments were amplified by PCR and cloned into cold shock expression vector, pColdII (Fig 1A), as described in the Materials and Methods section. The amino acid sequences of recombinant Pd-, P224-, and P145-ST are shown in S1–S3 Figs. All of the enzymes were expressed well in *E*. *coli* at low-temperature and purified with high purity by His-tag affinity chromatography using a Ni-NTA column (Fig 1B–1D). From 500 ml culture of transformed *E*. *coli* cells, 20 mg of Pd-ST, 20 mg of P224-ST, and 30 mg of P145-ST were purified.

### Enzyme activity comparisons for α(2,6)-sialylation of bi-antennary *N*-glycan

**Fig 2:**
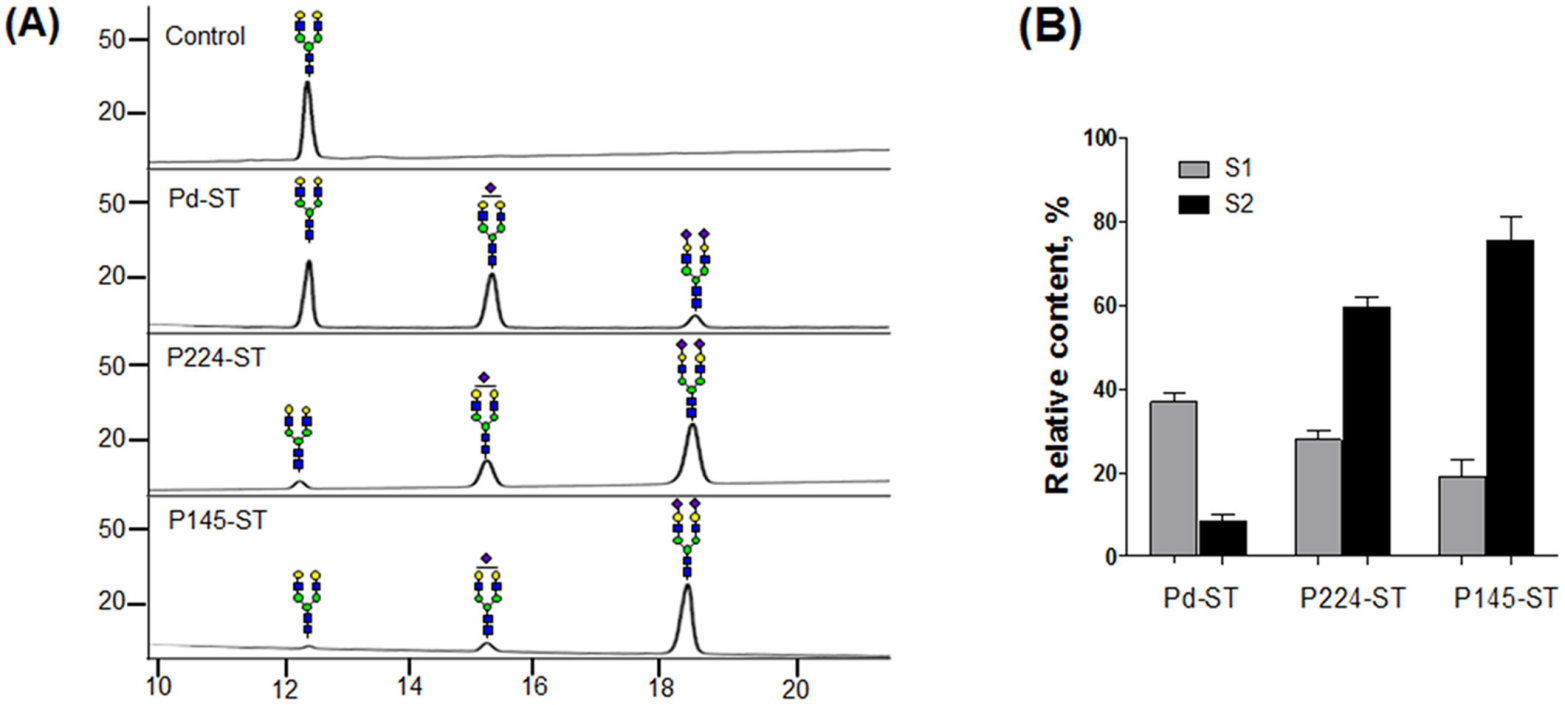
ST activity assays of bacterial α(2,6)-STs using G2 glycan as an accepting substrate. (A) The conversion of AA-labeled G2 glycan to sialylated glycans by Pd-, P224-, and P145-STs was analyzed by HPLC. The peaks of mono-sialylated (S1) and di-sialylated glycans (S2) as well as G2 glycan are indicated by symbol representation suggested by the Consortium for Functional Glycomics (http://www.functionalglycomics.org/). Blue square: GlcNAc, green circle: mannose, yellow circle: galactose, purple diamond: sialic acid. (B) Relative contents (%) of S1 (gray bar) and S2 glycan (black bar) were obtained by the calculation of integrated peak areas (100 × [The areas of corresponding glycan peaks]/[Total areas of all identified peaks]).

We compared the α(2,6)-ST activities of three purified enzymes (Pd-, P224-, and P145-ST) using an AA-labelled G2 glycan as an acceptor substrate: fluorescence-tag AA was conjugated to the glycan for detection in HPLC analysis. After the enzyme reactions, conversions to the sialylated forms S1 (Neu5Ac_1_Gal_2_Man_3_GlcNAc_4_) and S2 (Neu5Ac_2_Gal_2_Man_3_GlcNAc_4_) were analyzed using a Shodex Asaipack NH2P-50 amine column, which separates glycans based on charge and size (Fig 2A). In the tested condition for 30 min, P145-ST achieved the highest content of S2 glycan (76%) while Pd- and P224-ST made only 8.5% and 60% of S2 glycans, respectively (Fig 2B), which indicates the strongest ST activity of P145-ST. Steady-state kinetic parameters were also obtained (S4 Fig). The apparent K_m_ values of Pd-, P224-, and P145-ST for G2-AA substrate are 36.1 ± 4.9, 39.2 ± 4.7, and 27.8 ± 5.8 μM respectively. P145-ST has the highest V_max_/K_m_ value (6.3 nmol/min/mg/μM).

### Decreases of sialylated glycans in prolonged enzyme reactions

**Fig 3:**
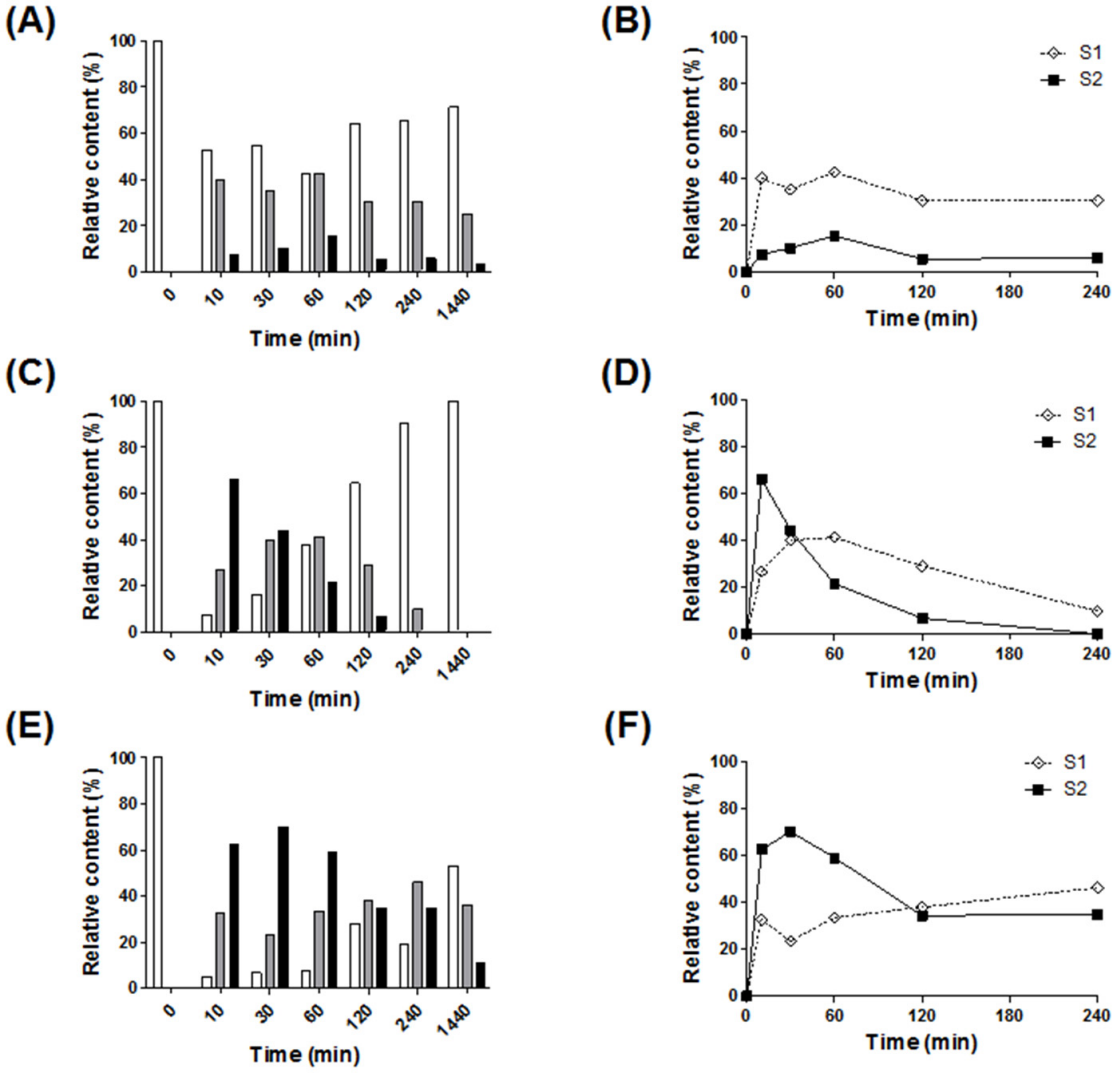
Prolonged incubation of bacterial α(2,6)-ST reaction decreased sialylated glycans. The time-courses of the enzyme reactions were performed at 30°C using Pd- (A and B), P224- (C and D), and P145-STs (E and F). In the bar graphs (A, C, and E), relative contents (%) of G2 (white bar), S1 (gray bar), and S2 (black bar) glycans are represented from 0–1,440 min, which were obtained by the calculation of integrated peak areas (100 × [The areas of corresponding glycan peaks]/[Total areas of all identified peaks]). In line graphs (B, D, and F), relative contents (%) of S1 (open diamond) and S2 (filled square) glycans are represented from 0–240 min.

During the analysis of bacterial α(2,6)-ST activities, we found, unexpectedly, that the relative contents of sialylated glycans decreased as the reactions were prolonged (Fig 3). In all three STs, the relative contents of di-sialylated S2 glycan show unimodal curves with a single maximum peak between 10–60 min (filled squares in Fig 3B, 3D and 3F), while mono-sialylated S1 glycan displays complex curve shapes depending on α(2,6)-STs (open diamonds in the same graphs). The S2 glycan curve in the P224-ST reaction reached a maximum peak with ~66% content in the shortest time (10 min) and then decreased to zero value by 240 min (Fig 3D). The Pd-ST reaction had the S2 glycan peak with the lowest maximum content value (~15%) at 60 min (Fig 3B). P145-ST had the highest maximum content of S2 glycan (~70%) at 30 min with a decrease to ~30% as the reaction was prolonged (Fig 3F). These results strongly suggest that all three α(2,6)-STs may have additional sialidase activities, which are more strongly induced as the reaction is prolonged.

### α(2,6)-Sialidase activity assay

**Fig 4:**
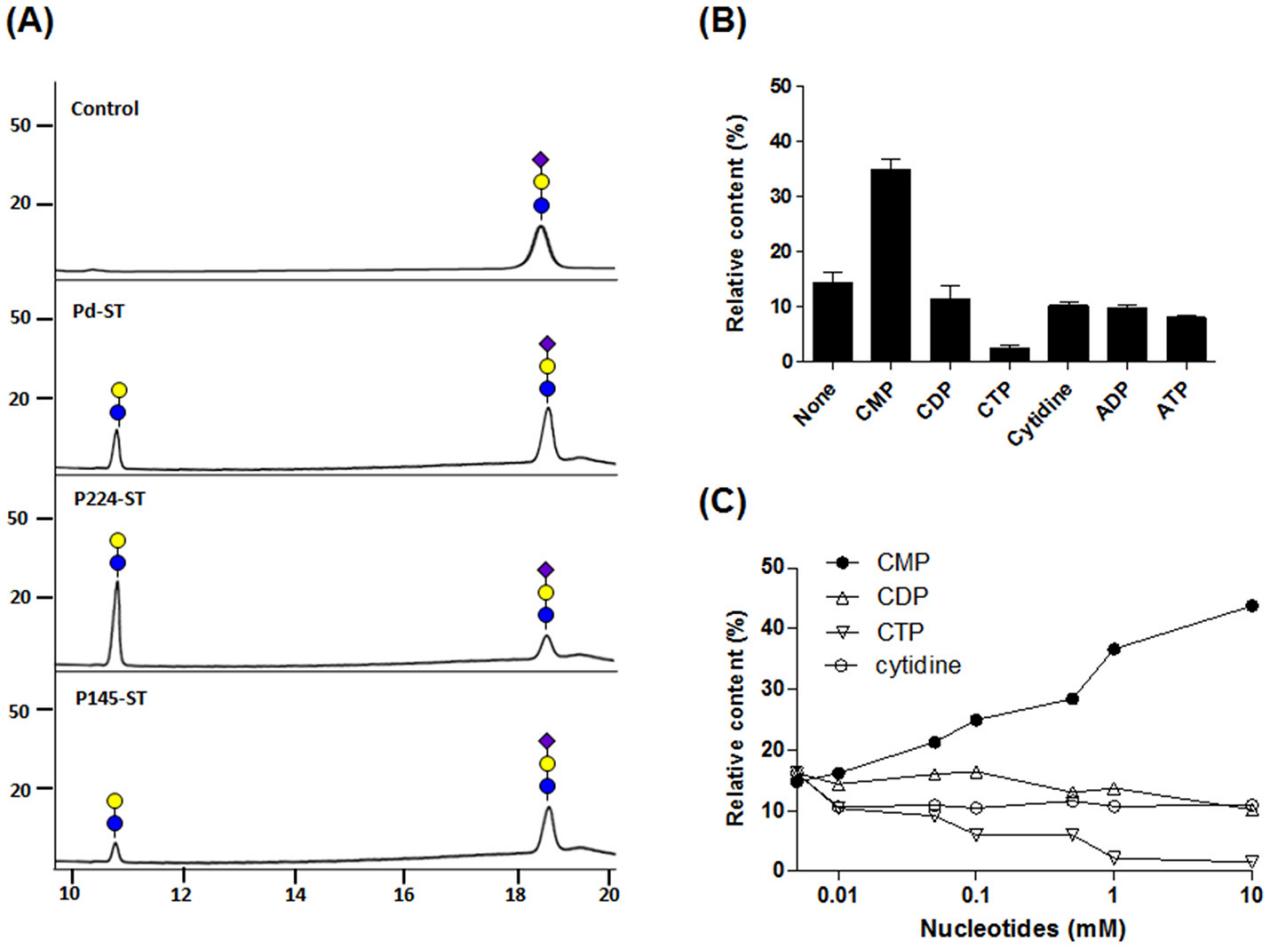
Sialidase activity analysis of bacterial α(2,6)-STs. (A) Removal of SA from AA-labeled 6’-sialyllactoses by Pd-, P224-, and P145-STs was analyzed using HPLC. Symbols for galactose and Neu5Ac are the same as in Fig 2, while glucose is represented by a blue circle. (B) The effects of nucleotides (CMP, CDP, CTP, cytidine, ADP, and ATP) on the α(2,6)-sialidase activity of P145-ST were examined. Reaction mixtures containing 1 mM of each nucleotide were incubated for 2 h. (C) Changes of P145-ST sialidase activities were also analyzed with addition of various concentrations (0.01, 0.05, 0.1, 0.5, 1, and 10 mM) of CMP (filled circle), CDP (triangle), CTP (reverse triangle), and cytidine (filled circle). The reaction mixtures were incubated for 2 h at 37°C. Relative contents (%) of lactose generated by sialidase activity were obtained by the calculation of integrated peak areas (100 × [The areas of lactose peaks]/[Total areas of lactose and sialyllactose peaks]).

Among three α(2,6)-STs, only Pd-ST had been proven experimentally to have α(2,6)-sialidase activity [14]. In this study, we examined whether P224- and P145-STs have sialidase activity using an AA-labeled 6`-sialyllactose as a substrate. After incubation with each of the bacterial α(2,6)-STs, HPLC analysis showed that some 6`-sialyllactose was converted to lactose (Fig 4A). In a 2 h reaction to compare them under the same condition, P224-ST showed the strongest sialidase activity (generation of ~70% lactose), while Pd- and P145-ST generated ~19% and ~13% lactose.

Cheng *et al*. showed that sialidase activity of Pd-ST was enhanced by the nucleotide product (CMP) of ST reaction although CMP was not essential component for sialidase activity [14]. Moreover, because the sialidase activities of all STs including Pd-ST appeared to be induced more strongly with increased incubation time of the reactions (bell-shaped curves of S2 glycan contents in Fig 3B, 3D and 3F), we investigated whether nucleotides including CMP affect the sialidase activity. As expected, the addition of CMP increased the sialidase activity whereas all other tested nucleotides including CDP, CTP, and cytidine showed decreasing effects to different degrees (Fig 4B for P145-ST and S5 Fig for Pd- and P224-STs). Sialidase activity of P145-ST increased proportionally as the concentration of CMP rose (Fig 4C): the content of lactose increased up to 43% at the highest tested concentration of CMP (10 mM). Although the sialidase activity of P145-ST decreased with CTP, CDP and cytidine, the degree and mode of their inhibitions were different. CTP and CDP inhibited sialidase activity in a concentration-dependent manner: especially, there was a great decrease in sialidase activity with CTP, which resulted in only 2% of lactose at 10 mM CTP. On the other hand, cytidine appeared to inhibit sialidase activity in an on-and-off mode.

### Inhibition of α(2,6)-sialidase activity by using AP

**Fig 5:**
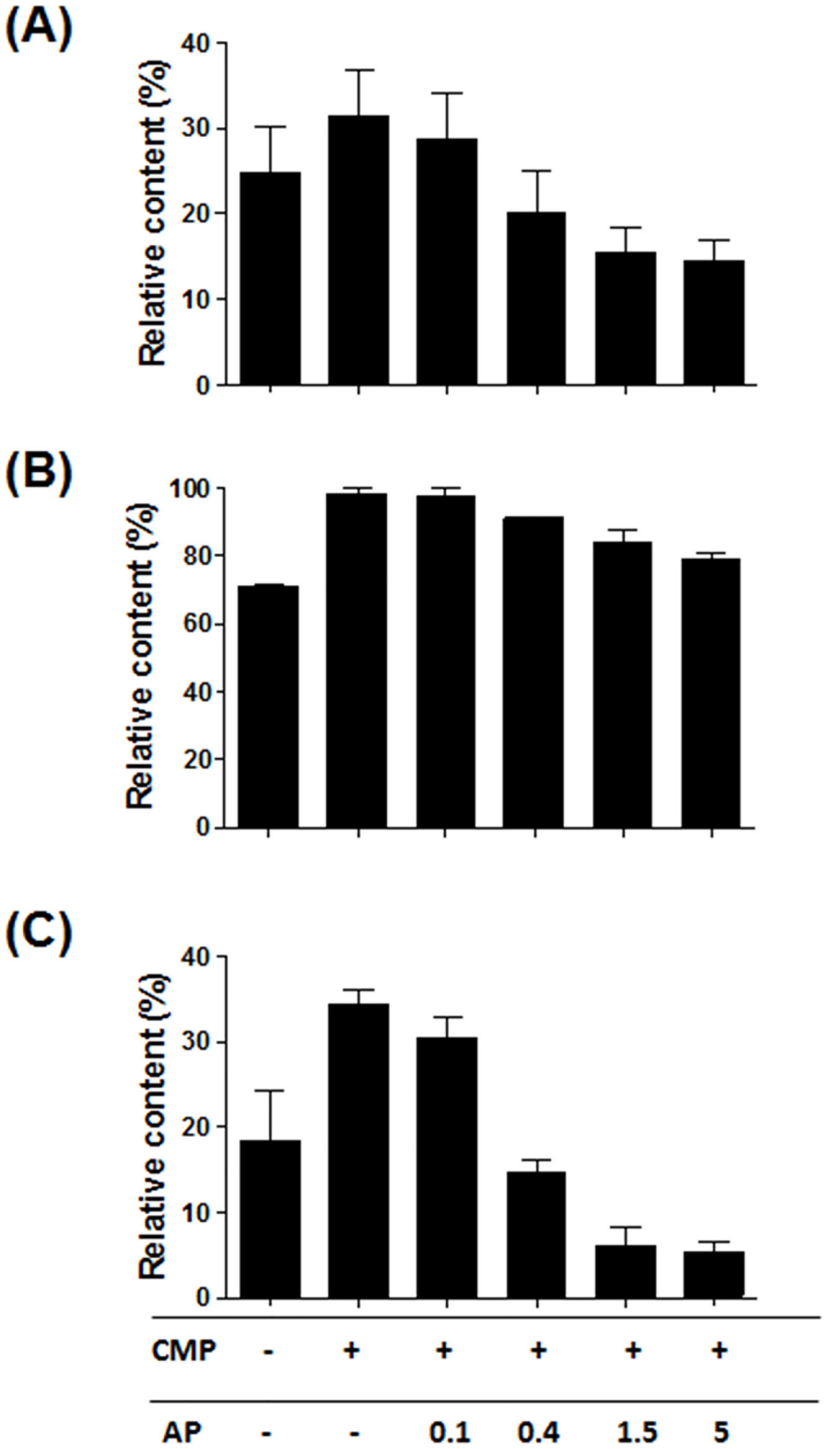
Inhibition of sialidase activity of bacterial α(2,6)-STs by AP. Sialidase activities of Pd- (A), P224- (B), and P145-STs (C) increased by 1 mM CMP addition were inhibited by supplemental addition of AP in a concentration dependent manner (0.1, 0.4, 1.5, and 5 U/μl). Relative contents (%) of lactose were calculated in the same way as in Fig 4.

It was deduced that AP treatment would decrease sialidase activity through the dephosphorylation of CMP to cytidine because the sialidase activities of bacterial α(2,6)-STs were increased by CMP and inhibited by cytidine. As expected, the increased sialidase activities from the addition of 1 mM CMP were inhibited by AP treatment (Fig 5). By increasing the amount of AP, the sialidase activities of Pd- and P145-STs were inhibited even more than for the reaction conditions without CMP addition. Especially, AP treatment with the P145-ST reaction mixture decreased the lactose content to 5%, which is almost 1/4 of that (19%) for the reaction without CMP addition. This indicates that the cytidine generated by AP treatment decreases sialidase activities. In the case of P224-ST, treatment of AP (up to 5 U/μl) did not decrease the lactose content to the level of the reaction without CMP addition, although AP still showed a concentration-dependent inhibitory effect. In summary, the sialidase activity of P145-ST was the lowest and could be inhibited further by AP treatment.

### Increase of α(2,6)-ST activity by addition of CMP-Neu5Ac and AP

**Fig 6:**
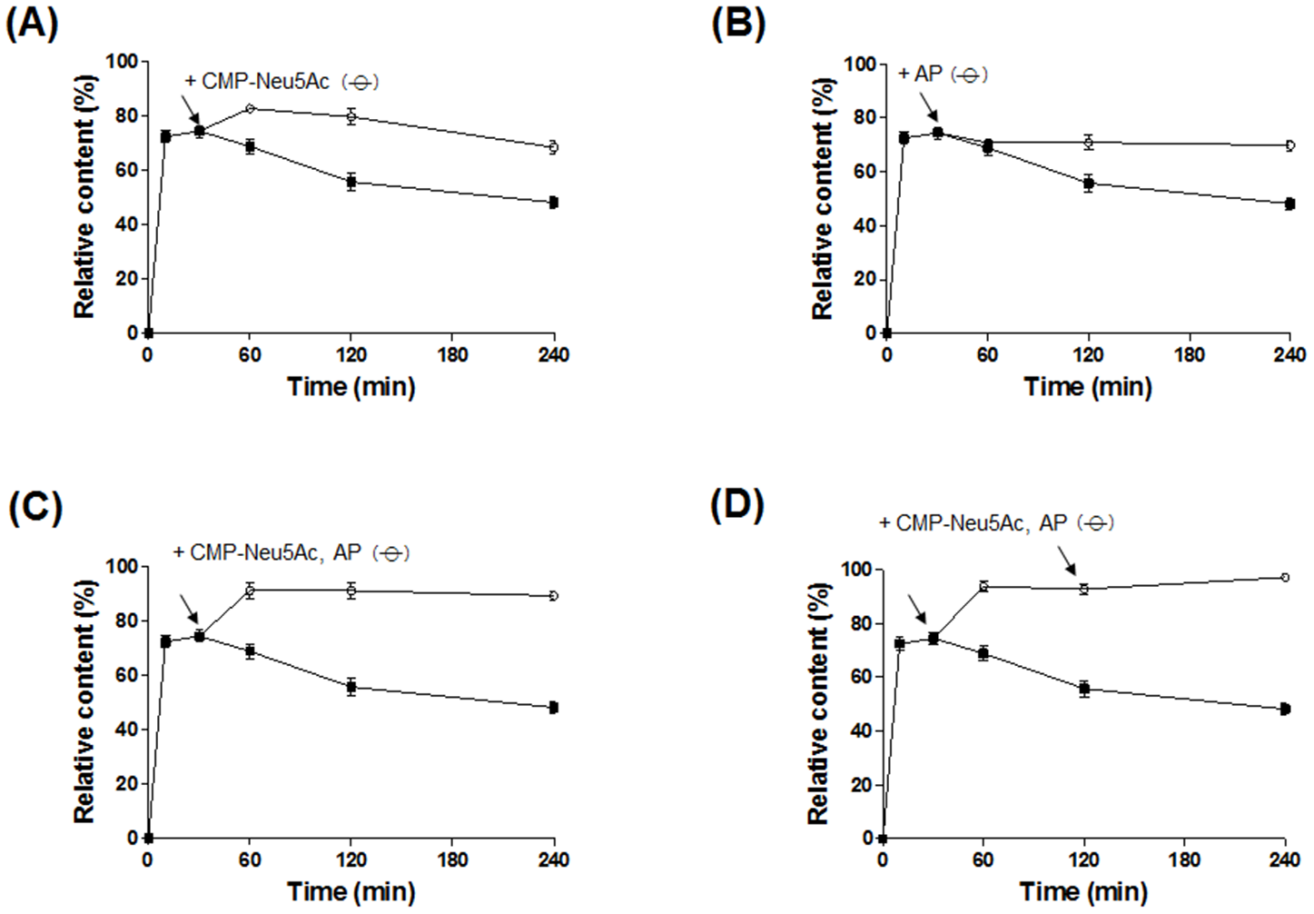
Optimized P145-ST reaction for increase of S2 glycan generation. For increase of S2 glycan content (%), additional CMP-NeuAc (A) or AP (B) was added 30 min after the beginning of the P145-ST reaction. Further increase of S2 glycan content was achieved by the simultaneous addition of CMP-NeuAc and AP at 30 min of the reaction (C). Through two sequential additions of both CMP-NeuAc and AP at 30 and 120 min of the reaction, relative content of S2 glycan increased up to 98% (D). Arrows indicate the time points for additions of CMP-NeuAc and/or AP. Open circles and filled squares represent the relative contents (%) of S2 glycan generated by the reactions with and without the supplemental addition respectively.

The content of di-sialylated S2 glycan reached a maximum at 30 min in the P145-ST reaction and decreased after that (filled squares in Figs 3F and 6). The supply of CMP-Neu5Ac to the reaction at 30 min led to a further increase in S2 glycan content up to 83% at 60 min, and then it started to decrease (open circles in Fig 6A). On the other hand, the addition of AP at 30 min prevented the decline of S2 glycan content, and the content level was maintained for the tested time (open circles in Fig 6B). With simultaneous addition of both CMP-Neu5Ac and AP to the reaction at 30 min, the S2 glycan content increased up to 92% and maintained that level for 240 min (open circles in Fig 6C). To further enhance the S2 glycan content, both CMP-Neu5Ac and AP were added to the reaction at 30 and 120 min, and this resulted in the S2 glycan content reaching 98% (open circles in Fig 6D). These results show that the α(2,6)-sialylation efficiency of the P145-ST reaction can be enhanced greatly through the supplementation of CMP-Neu5Ac and dephosphorylation of the generated CMP to cytidine by AP treatment.

**Fig 7:**
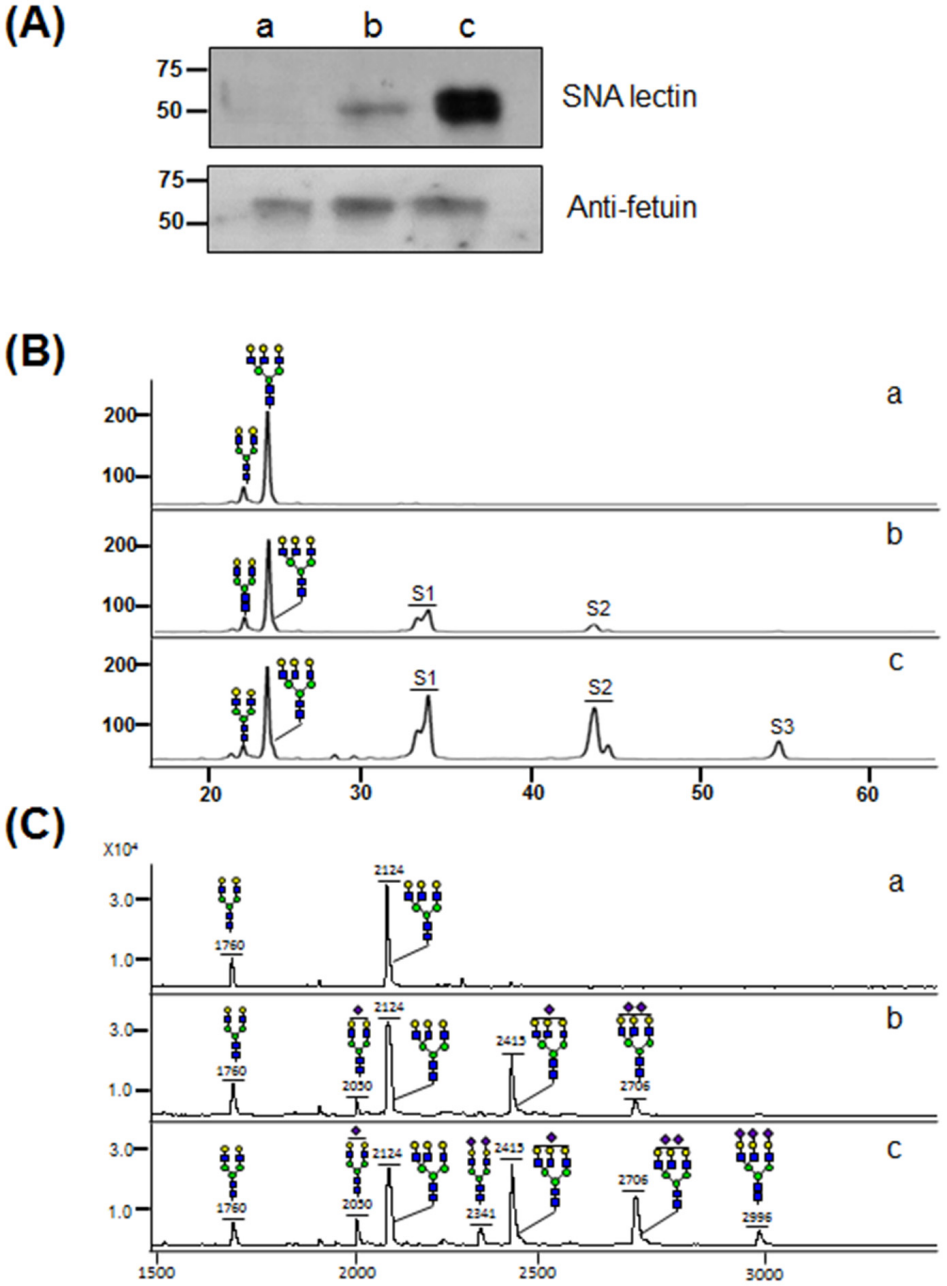
*In vitro* sialylation of asialofetuin by P145-ST. (A) Lectin blot assay was performed using SNA lectin for detection of α(2,6)-sialic acid of fetuin sialylated by P145-ST (upper panel). As a control experiment, immunoblot assay was also carried out using an anti-fetuin antibody (lower panel). *N*-glycans obtained from asialofetuins sialylated by P145-ST reaction were analyzed using HPLC (B) and MALDI-TOF (C). S1, S2, and S3 represent mono-, di-, and tri-sialylated glycans respectively. Symbols for glycans are the same as in Fig 2. Here, ‘a’: asialofetuin as “a”: asialofetuin as a control without ST reaction, “b”: sialylated asialofetuin in the reaction condition without supplemental addition, “c”: sialylated asialofetuin in the optimized condition with the supplemental addition of CMP-NeuAc and AP.

With application of the optimized P145-ST reaction condition including supplemental addition of CMP-Neu5Ac and AP, α(2,6)-sialylation of asialofetuin was enhanced greatly compared with the P145-ST reaction without the addition of CMP-Neu5Ac and AP (Fig 7). In lectin blot analysis using SNA lectin recognizing α(2,6)-linked SA, the optimized P145-ST reaction condition generated a much stronger band compared with the control reaction without supplementation (Fig 7A). The *N*-glycans of asialofetuin and sialylated fetuins from the P145-ST reaction were analyzed by HPLC (Fig 7B) and MALDI-TOF (Fig 7C). In HPLC analysis, AA-labeled glycans were separated by an amine column, based on the charge and size (negative and larger glycans were eluted later). This enabled the glycan peaks to be classified into four groups, neutral, mono- (S1), bi- (S2), and tri- (S3) glycan, which were also verified by checking the masses of the collected peaks using MALDI-TOF (data not shown). P145-ST reaction without addition of CMP-Neu5Ac and AP generated only small amounts of S1 (33%) and S2 glycans (10%). In contrast, with the fetuin sialylated in the optimized P145-ST reaction condition, S3 glycan (8%) was observed as well as greatly increased amounts of S1 (36%) and S2 glycans (29%). Glycan mass profiles also show that the optimization of the P145-ST reaction increases the intensities of sialylated glycan peaks (Fig 7C). Notably, fully sialylated di-antennary (Neu5Ac_2_Gal_2_Man_3_GlcNAc_4_) and tri-antennary glycans (Neu5Ac_3_Gal_3_Man_3_GlcNAc_5_) without exposed galactose residues were only detected in the optimized reaction condition. All these results clearly indicate that the P145-ST reaction sialylates glycoproteins with greatly increased efficiency upon the supplemental addition of CMP-Neu5Ac and AP.

## Discussion

Bacterial α(2,6)-STs have several advantages, including easy and economic expression in *E*. *coli* and broader acceptor substrate specificity, compared with eukaryotic α(2,6)-STs, which require an expensive expression system such as insect or mammalian cells [4,6]. In this study, we compared directly three bacterial α(2,6)-STs recombinantly expressed in *E*. *coli* in order to determine which one is the best for α(2,6)-sialylation of glycoproteins. Currently, four bacterial α(2,6)-STs have been found and characterized from *P. damsela [7]*, *Photobacteriium* sp. JT-ISH-224 [10,15], and *P*. *leiognathi* JT-SHIZ-145 [8] and JT-SHIZ-119 [9], all of which belong to the genus *Photobacterium*. Because α(2,6)-STs from *P*. *leiognathi* JT-SHIZ-145 and JT-SHIZ-119 display 95% of amino acid sequence identity (S2 Table), we only chose P145-ST and compared the activities of three STs (together with Pd- and P224-STs) using a G2 glycan, which is the common *N*-glycan substrate found in glycoprotein. This comparison study indicates that P145-ST has the highest activity to generate S2 glycan from the G2 substrate (Fig 2).

The contents of di-sialylated S2 glycan in bacterial α(2,6)-ST reactions first increased to a maximum value and then decreased as the reaction time was prolonged (Fig 3). We assumed that this tendency resulted from the inherent sialidase activities of bacterial α(2,6)-STs. There are conflicting reports about the sialidase activities of these STs. Mine *et al*. reported that α(2,6)-ST derived from *P*. *leiognathi* strain JT-SHIZ-119 has both ST and sialidase activities whereas Pd-, P224- and P145-STs do not [9]. On the other hand, Cheng *et al*. reported that Pd-ST has α(2,6)-sialidase activity that can be enhanced by CMP and used for the α(2,6)-trans-sialidase reaction [14]. In our experiment, all of the three STs displayed sialidase activities (Fig 4A). Among them, P224-ST showed the highest sialidase activity. Its strong sialidase activity can explain the observation that the relative content of S2 glycan in the P224-ST reaction reached the maximum value at the shortest time (10 min) and then decreased most rapidly to the zero level (Fig 3C and 3D).

The decreases of S2 glycan content at later times of the ST reactions raised the possibility that free CMP generated from CMP-Neu5Ac by the transfer of SA to an acceptor increases sialidase activity. This possibility was more strengthened by the previous observation that the sialidase activity of Pd-ST was enhanced by CMP [14]. In accord with this expectation, CMP increases the sialidase activities in a concentration-dependent manner. From this observation, we deduced that the conversion of CMP to cytidine, which can be easily achieved by AP treatment, would lead to inhibition of sialidase activity. As expected, AP treatment inhibits the sialidase activities of the three STs and the inhibitory effect is most prominent in the P145-ST reaction (Fig 5). This AP treatment strategy was applied successfully to prevent the decrease of S2 glycan content at later times of the P145-ST reaction (Fig 6B). Notably, while CMP-Neu5Ac addition increased the content of S2 glycan (Fig 6A), AP treatment prevented only the decrease in the content. This suggests that the enhancement of α(2,6)-sialylation by AP treatment is achieved mainly through the inhibition of inherent sialidase activity. Furthermore, simultaneous additions of AP and acceptor substrate (CMP-Neu5Ac) produced a synergistic effect: there was a great increase in the level of S2 glycan content and this level was maintained throughout the remainder of the tested period without any decrease (Fig 6C and 6D). This series of experiments clearly indicates that AP treatment increased the content of S2 glycan by inhibiting the inherent sialidase activity of bacterial α(2,6)-STs.

It is noteworthy that CTP and CDP as well as cytidine inhibit sialidase activities (Fig 4C). Although a strategy to convert CMP to CDP or CTP by phosphorylation may enhance the efficiency of α(2,6)-ST through inhibition of inherent sialidase activity, it is not a favorable solution compared with simple AP reaction because conversions to CDP and CTP require enzyme reaction with input of energy. As an alternative, we speculate that sugar nucleotide (CMP-Neu5Ac) regeneration methods [16,17] will increase the efficiency of bacterial α(2,6)-ST reactions by converting the generated CMP to CMP-Neu5Ac through a series of enzyme reaction. Although this CMP-Neu5Ac regeneration strategy has many advantages such as its economic reuses, it is disappointedly not compatible with the strategy using AP treatment. However, because CMP-Neu5Ac regeneration method usually requires more than four additional enzymes and energy inputs for this complex enzyme reactions [16,17], simple addition of AP and CMP-Neu5Ac would be more convenient and easier solution for increase of α(2,6)-sialylation efficiency.

We selected P145-ST to test α(2,6)-sialylation of asialofetuin because it has more advantages compared to Pd- and P224-ST; (1) P145-ST has the highest activity to generate S2 from G2 glycan (Fig 2), (2) its sialidase activity was the most efficiently inhibited by the strategy using AP treatment (Fig 3), and (3) it can convert most of the G2 populations to S2 (98%) through the supplemental additions of CMP-Neu5Ac and AP (Fig 6), which was not achieved by Pd- and P224-ST. The optimized P145-ST reaction with additions of both CMP-Neu5Ac and AP was applied successfully for α(2,6)-sialylation of asialofetuin with a great increase of α(2,6)-sialylated glycans compared with the non-optimized reaction without additions (Fig 7). Notably, the non-optimized P145-ST reaction was extremely inefficient for the generation of multi-sialylated glycans: only ~10% S2 and no S3 glycans were observed. In contrast, the optimized condition generated ~29% S2 (three-fold increase) and 8% S3 glycans. Moreover, fully sialylated di- and tri-antennary glycans without exposed galactose residues were only detected in the glycan mass profile of asialofetuin sialylated in the optimized condition. It may be that inherent sialidase activity of P145-ST removes SA preferentially from multi-silaylated glycans. Collectively, these results suggest that inhibition of sialidase activity by AP treatment is an essential requirement for increased efficiency of α(2,6)-sialylation and the generation of multi-sialylated glycoproteins.

We also applied the optimized P145-ST reaction for generation of the IgG Fc fragment attached with α(2,6)-sialylated glycan, which is known to have anti-inflammatory efficacy for treatment of rheumatoid arthritis [3]. However, the optimized P145-ST reaction (as well as the non-optimized one) was not very effective for adding α(2,6)-SA to the galactosylated *N*-glycan at Asn297 of Fc (data not shown). It has been reported that sialylation of Fc *N*-glycan is highly challenging and therefore requires a fairly large amount of highly active eukaryotic α(2,6)-ST (ST6Gal1) [4,18]. We speculate that bacterial α(2,6)-ST including P145-ST might be more affected by steric hindrance of the glycan attachment region of Fc because free *N*-glycan released from Fc by PNGase F treatment was almost completely sialylated by the optimized P145-ST reaction (data not shown).

In summary, we compared the activities of bacterial α(2,6)-STs expressed in *E*. *coli* and found that P145-ST is the most effective to generate di-sialylated glycan from a galactosylated *N*-glycan. To overcome inherent sialidase activity induced by free CMP generated during the ST reaction, AP treatment was used successfully to inhibit the sialidase activity by dephosphorylation of CMP to cytidine. The α(2,6)-siaylation efficiency of P145-ST reaction is increased greatly by supplemental addition of both CMP-Neu5Ac and AP, and this reaction can be used to generate a multi-sialylated glycoprotein. As an alternative to eukaryotic α(2,6)-STs, which requires an expensive expression host, the optimized P145-ST reaction has a great promise as an economic method for glycan synthesis and glyco-conjugate remodeling.

## Supporting Information

S1 Fig Nucleotide and amino acid sequences of the recombinant Pd-ST. Underlined sequences are derived from pColdII vector.
(TIF)

S2 Fig Nucleotide and amino acid sequences of the recombinant P224-ST. Underlined sequences are derived from pColdII vector.
(TIF)

S3 Fig Nucleotide and amino acid sequences of the recombinant P145-ST. Underlined sequences are derived from pColdII vector.
(TIF)

S4 Fig Steady-state kinetic parameters of bacterial α(2,6)-STs. Kinetic parameters of Pd-, P224-, and P145-STs were obtained by fitting the experimental data to the Michaelis-Menten equation using nonlinear regression analysis.
(TIF)

S5 Fig Effects of nucleotides on the α(2,6)-sialidase activity of Pd- and P224-STs. Relative contents (%) of lactose generated by sialidase activity of Pd- (A) and P224-STs (B) were obtained from 2 hr reaction with the addition of 1 mM of CMP, CDP, CTP, cytidine, ADP, or ATP through the calculation of integrated peak areas (100 × [The areas of lactose peaks]/[Total areas of lactose and sialyllactose peaks])
(TIF)

S1 Table PCR Primers used in this study. (DOCX)

S2 Table Amino acid sequence identities and similarities among bacterial α(2,6)-STs. (DOCX)
