# Supplementary figures and images for: Enhanced Bacterial α(2,6)-Sialyltransferase Reaction through an Inhibition of Its Inherent Sialidase Activity by Dephosphorylation of Cytidine-5'-Monophosphate
Source: PLoS One. 2015 Jul 31;10(7):e0133739. doi: 10.1371/journal.pone.0133739 (PMC4521712; doi:10.1371/journal.pone.0133739)

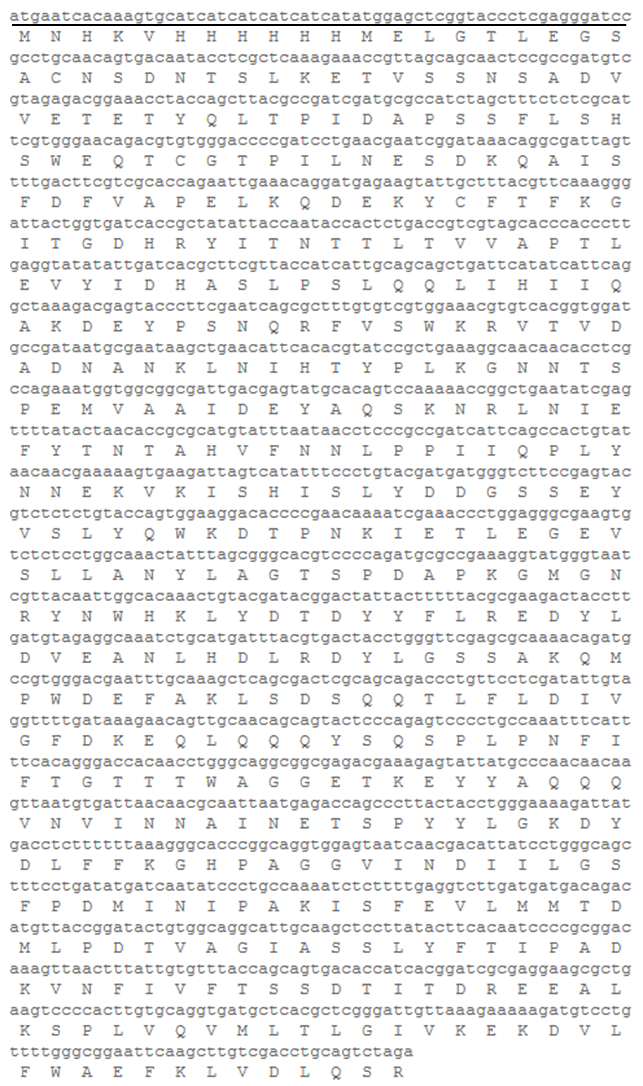

Supplement: S1 Fig — Underlined sequences are derived from pColdII vector. (TIF) [file pone.0133739.s001.tif]

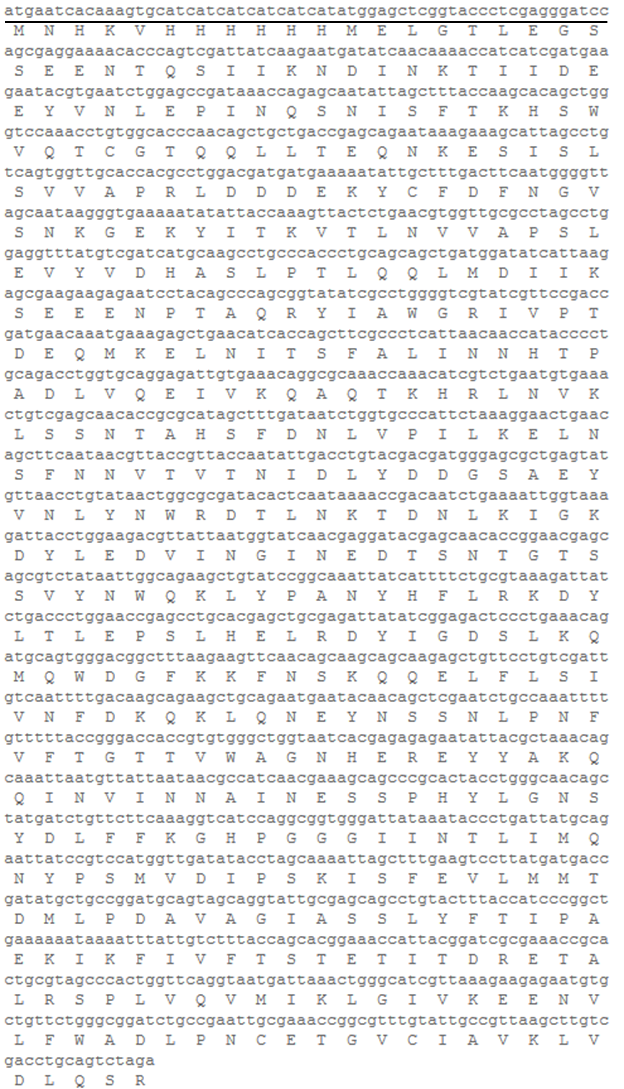

Supplement: S2 Fig — Underlined sequences are derived from pColdII vector. (TIF) [file pone.0133739.s002.tif]

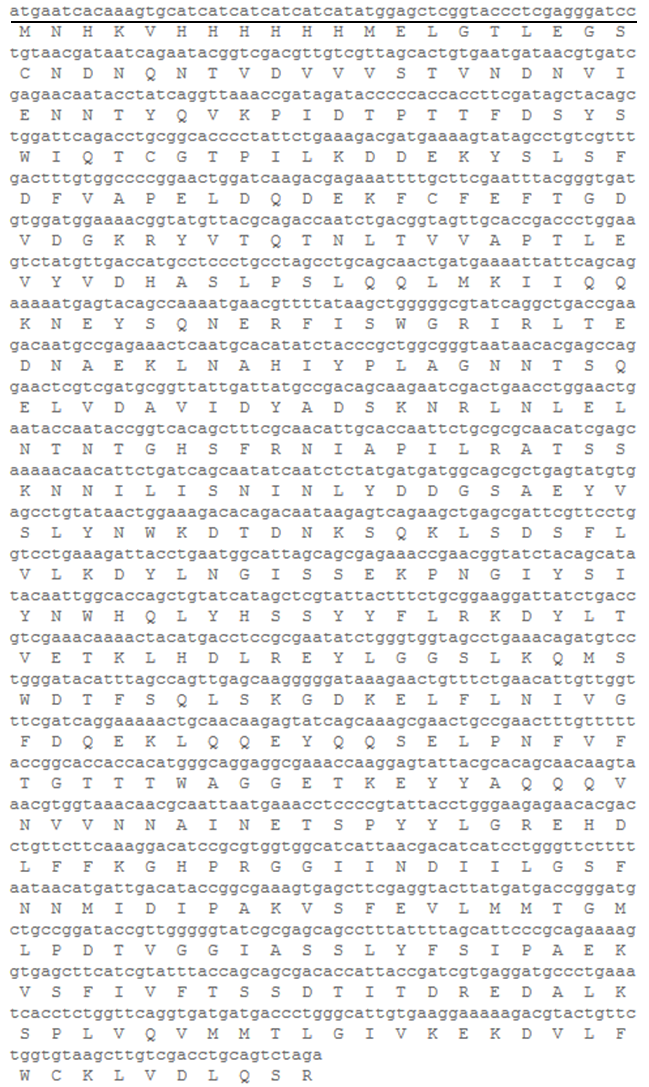

Supplement: S3 Fig — Underlined sequences are derived from pColdII vector. (TIF) [file pone.0133739.s003.tif]

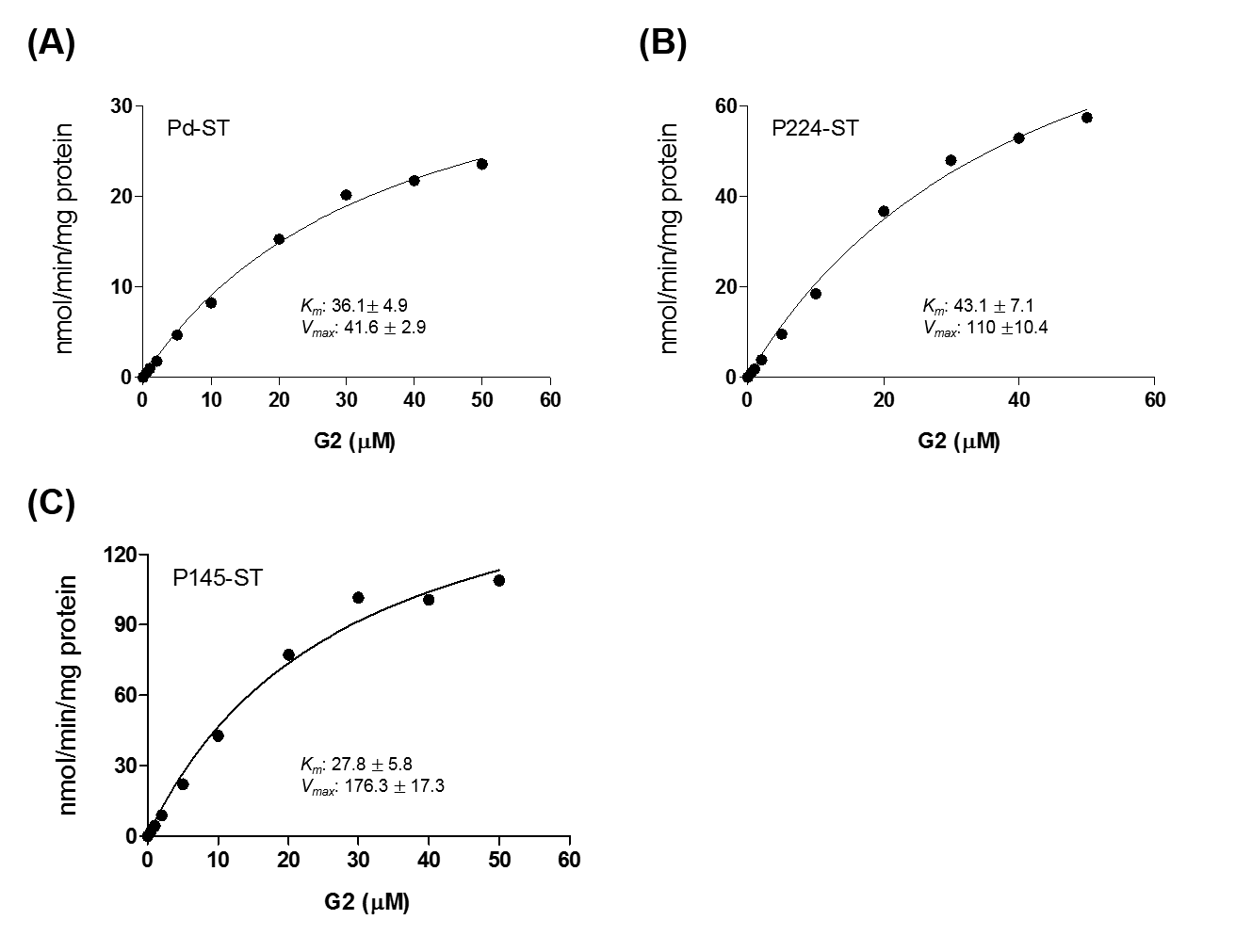

Supplement: S4 Fig — Kinetic parameters of Pd-, P224-, and P145-STs were obtained by fitting the experimental data to the Michaelis-Menten equation using nonlinear regression analysis. (TIF) [file pone.0133739.s004.tif]

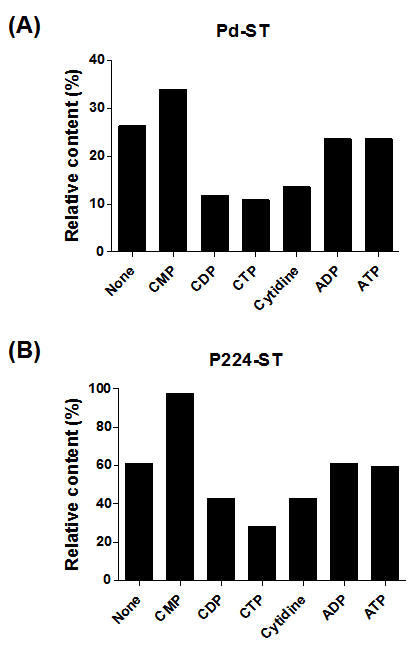

Supplement: S5 Fig — Relative contents (%) of lactose generated by sialidase activity of Pd- (A) and P224-STs (B) were obtained from 2 hr reaction with the addition of 1 mM of CMP, CDP, CTP, cytidine, ADP, or ATP through the calculation of integrated peak areas (100 × [The areas of lactose peaks]/[Total areas of lactose and sialyllactose peaks]) (TIF) [file pone.0133739.s005.tif]
