# Supplementary material for: Enhanced Bacterial α(2,6)-Sialyltransferase Reaction through an Inhibition of Its Inherent Sialidase Activity by Dephosphorylation of Cytidine-5'-Monophosphate
Source: PLoS One. 2015 Jul 31;10(7):e0133739. doi: 10.1371/journal.pone.0133739 (PMC4521712; doi:10.1371/journal.pone.0133739)
Supplement: S1 Table — (DOCX) [file pone.0133739.s006.docx]

**S1 Table. PCR Primers used in this study.**

| **Primers** | **Sequence**^a^ |
| --- | --- |
| Pd ST_F | GCGGATCCGCCTGCAACAGTGACAATACCTCG |
| Pd ST_R | GCGAATTCTTACGCCCAAAACAGGACATCTTTTTC |
| P224 ST_F | GCGGATCCAGCGAGGAAAACACCCAGTCG |
| P224 ST_R | GCAAGCTTAACGGCAATACAAACGCCGGTTTC |
| P145 ST_F | GCGGATCCTGTAACGATAATCAGAATACGG |
| P145 ST_R | GCAAGCTTACACCAGAACAGTACGTCTTTTTC |

^a^Sequences for restriction enzymes are underlined.
