# Supplementary material for: Enhanced Bacterial α(2,6)-Sialyltransferase Reaction through an Inhibition of Its Inherent Sialidase Activity by Dephosphorylation of Cytidine-5'-Monophosphate
Source: PLoS One. 2015 Jul 31;10(7):e0133739. doi: 10.1371/journal.pone.0133739 (PMC4521712; doi:10.1371/journal.pone.0133739)
Supplement: S2 Table — (DOCX) [file pone.0133739.s007.docx]

**S2 Table. Amino acid sequence identities and similarities among bacterial α(2,6)-STs.**

|  | Pd-ST | P224-ST | P145-ST | P119-ST |
| --- | --- | --- | --- | --- |
| Pd-ST |  | 74 | 81 | 81 |
| P224-ST | 55 |  | 75 | 77 |
| P145-ST | 67 | 55 |  | 96 |
| P119-ST | 67 | 57 | 95 |  |

**Similarity**

**Identity**
